# Assessment of violet-blue color formation in *Phalaenopsis* orchids

**DOI:** 10.1186/s12870-020-02402-7

**Published:** 2020-05-12

**Authors:** Che-Yu Liang, Krishna Preethi Rengasamy, Li-Min Huang, Chia-Chi Hsu, Mei-Fen Jeng, Wen-Huei Chen, Hong-Hwa Chen

**Affiliations:** 1 Department of Life Sciences, National Cheng Kung University, Tainan, 701 Taiwan; 2 Orchid Research and Development Center, National Cheng Kung University, Tainan, 701 Taiwan; 3 Nantou City, Taiwan

**Keywords:** Delphinidin, *DgF3’5’H*, Metal ions, Orchids, *PeF3’H*, pH, *PhF3’5’H*, *Phalaenopsis*, Violet-blue

## Abstract

**Background:**

*Phalaenopsis* represents an important cash crop worldwide. Abundant flower colors observed in *Phalaenopsis* orchids range from red-purple, purple, purple-violet, violet, and violet-blue. However, violet-blue orchids are less bred than are those of other colors. Anthocyanin, vacuolar pH and metal ions are three major factors influencing flower color. This study aimed to identify the factors causing the violet-blue color in *Phalaenopsis* flowers and to analyze whether delphinidin accumulation and blue pigmentation formation can be achieved by transient overexpression of heterologous *F3’5’H* in *Phalaenopsis*.

**Results:**

Cyanidin-based anthocyanin was highly accumulated in *Phalaenopsis* flowers with red-purple, purple, purple-violet, and violet to violet-blue color, but no true-blue color and no delphinidin was detected. Concomitantly, the expression of *PeF3’H* (*Phalaenopsis equestrsis*) was high, but that of *PhF3’5’H* (*Phalaenopsis* hybrid) was low or absent in various-colored *Phalaenopsis* flowers. Transient overexpression of *DgF3’5’H* (*Delphinium grandiflorum*) and *PeMYB2* in a white *Phalaenopsis* cultivar resulted a 53.6% delphinidin accumulation and a novel blue color formation. In contrast, transient overexpression of both *PhF3’5’H* and *PeMYB2* did not lead to delphinidin accumulation. Sequence analysis showed that the substrate recognition site 6 (SRS6) of PhF3’5’H was consistently different from DgF3’5’Hs at positions 5, 8 and 10. Prediction of molecular docking of the substrates showed a contrary binding direction of aromatic rings (B-ring) with the SRS6 domain of DgF3’5’H and PhF3’5’H. In addition, the pH values of violet-blue and purple *Phalaenopsis* flowers ranged from 5.33 to 5.54 and 4.77 to 5.04, respectively. Furthermore, the molar ratio of metal ions (including Al^3+^, Ca^2+^ and Fe^3+^) to anthocyanin in violet-blue color *Phalaenopsis* was 190-, 49-, and 51-fold higher, respectively, than those in purple-color *Phalaenopsis*.

**Conclusion:**

Cyanidin-based anthocyanin was detected in violet-blue color *Phalaenopsis* and was concomitant with a high pH value and high molar ratio of Al^3+^, Ca^2+^ and Fe^3+^ to anthocyanin content. Enhanced expression of delphinidin is needed to produce true-blue *Phalaenopsis*.

## Background

*Phalaenopsis* hybrids are among the most popular orchids in flower markets because of their long-lived and spectacular flowers. One of the special agronomic traits for *Phalaenopsis* is their abundant flower color, including white, yellow and red-purple. For the red-purple series, the flower colors range from red-purple, purple, purple-violet, violet and violet-blue (Additional file 1) according to the Royal Horticultural Society Color Chart (RHSCC). In Taiwan, red-purple flowers are the most popular because of the color’s association with happiness. However, violet-blue *Phalaenopsis* flowers are rarely seen in orchid markets mainly because of the difficulty in breeding blue-color flowers, few native species with blue flowers, short-lived flowers, small flower size, and easy color fading (Additional file 2). The earliest breeding of a blue flower cultivar of *Phalaenopsis*, recorded by the RHSCC, was named *P*. Kenneth Schubert, in 1963. After years of breeding, *Phalaenopsis* still lacks a true-blue color flower, and violet-blue is the bluest color in *Phalaenopsis* flowers. Hence, to breed a *Phalaenopsis* flower with blue color in addition to other stamen horticultural traits is desired and highly expected from both orchid breeders and consumers.

Flower colors are important for plants to be able to attract their pollinators. Blue is a beautiful and attractive color in flowers. However, there are few wild plants with blue flowers as compared with those with red flowers possibly because of the co-evolution of flower color and visual capture of their pollinators [1].

The three major classes of plant pigments involve the distinct chemical structures of betalains, carotenoids and anthocyanins [2]. Anthocyanin, one class of flavonoids soluble in water, is synthesized in the cytosol and localized in vacuoles. Seven core structural genes are well known to encode enzymes that catalyze the biosynthesis of anthocyanins [3]. In the biosynthesis of flavonoid and anthocyanin pigments, chalcone synthase is the first step that catalyzes sequential condensations with three acetate units from malonyl-CoA and ρ-coumaroyl-CoA. The result is naringenin chalcone (2′,4,4′,6′-tetrahydroxychalcone) and 6′-deoxychalcon (2′,4,4′-trihydroxychalcone) which are then catalyzed by chalcone isomerase via stereospecific cyclization to form naringenin (N) and liquiritigenin, respectively. Flavanone-3-hydroxylase (F3H) converts the flavonones (naringenin and eriodictyol) to dihydroflavonols (dihydrokaempferol [DHK]). Flavonoid-3′- hydroxylase (F3’H) and flavonoid-3′,5′-hydroxylase (F3’5’H) are functional flavonoid B-ring hydroxylation and are required for biosynthesis of flavones, flavanones, flavonols and anthocyanins. With anthocyanins, F3’H is needed for the formation of cyanidins and F3’5’H for the formation of delphinidins for red and blue colors, respectively. Both F3’H and F3’5’H compete for the common precursors of N and DHK. Dihydroflavonol-4-reductase (DFR) reduces dihydroflavonols (DHK, dihydroquercetin and dihydromyricetin) to leucoanthocyanidins (leucopelargonidin, leucocyanidin and leucodelphinidin). Anthocyanidin synthase (ANS) is involved in a crucial step of anthocyanin formation because it catalyzes the oxidation of colorless leucoanthocyanidin to the precursor of anthocyanidins. Glycosylation of a hydroxyl group at the C-3 position of anthocyanidins is the primary modification step needed to stabilize anthocyanins and is required for further modifications. UDP-glucose: flavonoid-3-0-glucosyl transferase (3GT) glycosylates anthocyanidins and flavonols on the 3 position to produce anthocyanins [4].

Previous studies showed many commercial horticultural plants without blue hue flowers due to lack of F3’5’H. Molecular approaches have been used to create blue-color flowers in rose [5], carnation [6, 7] and chrysanthemum by ectopic overexpression of F3’5’H [8, 9]. Chrysanthemums (*Chrysanthemum morifolium* Ramat.) without blue-, violet- or purple-flowered cultivars are due to the absence of the F3’5’H gene that encodes the key enzyme for delphinidin-based anthocyanin biosynthesis [9]. The flower colors of transgenic plants with heterologous F3’5’H overexpression produce delphinidin-based anthocyanins and change from a red-purple to a purple-violet hue [9]. Thus, F3’5’H may compete with F3’H for their common substrates, to lead to the production of dephinidin. Regulation of F3’5’H gene expression in the delphinidin-based anthocyanin biosynthesis pathway could be a successful strategy for high production of delphinidin-based anthocyanins and blue hue in *Phalaenopsis* flowers.

Different levels of vacuolar pH could cause a color shift in flowers with the same anthocyanin compounds. Flowers with the same anthocyanin compounds can have a color change with diverse levels of vacuolar pH. For example, an increase in pH from 6.6 to 7.7 due to the polyacylation of peonidin glycoside cause blue coloration in the corolla of morning glory [10]. In addition, both *InNHX1* and *InNHX2* translate proteins responsible for transporting K^+^/Na^+^ into the vacuoles in morning glory, and produce weakly alkaline vacuoles at the flowering stage [11–13]. Na^+^(K^+^)/H^+^ exchanger (NHX) drives a vacuolar pH increase during flower opening, causing a color shift from red to blue [11]. In both mutants of V-type ATPase, *ph 5* and *ph 1*, vacuolar acidification was reduced and caused purple-colored petunia [14, 15]. Blue color development of metalloanthocyanins obtained from different blue flower petals has been found. Mg^2+^ and Fe^3+^ are essential for blue coloration in blue poppy flower [16, 17]. Fe^3+^ and Al^3+^ may affect blue coloration in the blue cornflower *Centaurea cyanus* [18]. The molar ratio of vacuolar metal ions, flavones and anthocyanin is 2:6:6 and forms a supermolecular complex, with metalloanthocyanin acting as a conclusive factor, causing blue blooms [16]. Therefore, the content of specific metal ions is a crucial factor influencing blue coloration in flowers.

The R2R3-MYB transcription factors are crucial in influence on the spatial and temporal patterning of anthocyanins in many flowers [19–21]. In *Phalaenopsis*, three PeMYBs, including PeMYB2, PeMYB11, and PeMYB12 involve in the different pigmentation patterning of full-red pigmentation, red spots, and venation, respectively [22]. Transient overexpression of *PeMYB2* in white-color *Phalaenopsis* flower, which has little or no expression of *PeMYB2*, causes red pigmentation and anthocyanin accumulation and stimulate the expression of downstream structural genes, *PeF3H*, *PeDFR*, and *PeANS* [22].

Here, we aimed to study the mechanisms in the formation of violet-blue color in *Phalaenopsis* flower. In addition, we tested the initial assumption framework on converting the white-color orchids to blue hue by transient overexpression of *PeMYB2* and heterologous *F3’5’H* as well as knockdown of endogenous *F3’H* in *Phalaenopsis* by using virus-induced gene silencing (VIGS) to access whether delphinidin could accumulate*.* This study will set the groundwork for molecular breeding of *Phalaenopsis* cultivars with novel blue color pigmentation.

## Results

### Cyanidin-based anthocyanin is highly accumulated in *Phalaenopsis* spp. of various colors

**Fig. 1:**
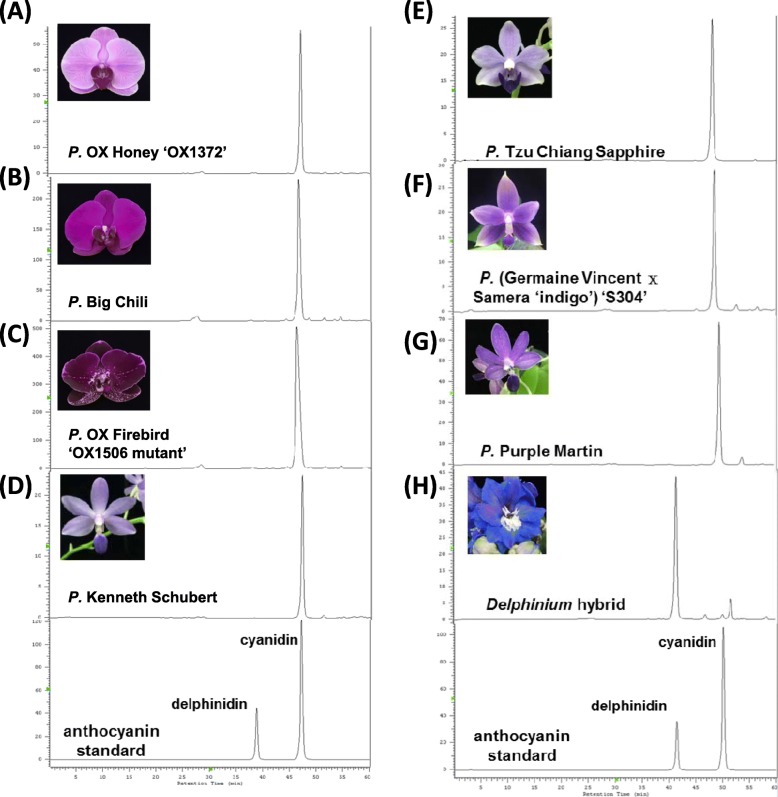
HPLC analysis of anthocyanin compound. Various flower colors of *Phalaenopsis* were used to determine the types of anthocyanin in various flower colors of *Phalaenopsis*, including (**a**) *P.* OX Honey ‘OX1372’, (**b**) *P*. Big Chile, (**c**) *P*. OX Firebird ‘OX1506 mutant’, (**d**) *P*. Kenneth Schubert, (**e**) *P*. Tzu Chiang Sapphire, (**f**) *P*. (Germaine Vincent x Samera ‘indigo’) ‘S304’, (**g**) *P*. Purple Martin, and (**h**) *Delphinium* hybrid*. Delphinium* was included as a control group. Anthocyanin compounds of delphinidin and cyanidin were also included to show the peaks of standards

To examine whether anthocyanins differentially accumulated in *Phalaenopsis*, anthocyanins were extracted and analyzed by using HPLC. Pure compounds of both cyanidin and delphinidin were used as standards (Fig. 1). Cyanidin was the major and only anthocyanin compound detected in *Phalaenopsis* of different colors (Fig. 1a-g), including purple *P*. OX Honey ‘OX1372’, purple-violet *P*. Big Chili, dark purple *Phal*. OX Firebird ‘OX1506 mutant’, violet-blue *Phal*. Kenneth Schubert, violet-blue *P. tau* Chiang Sapphire, violet *P*. (Germaine Vincent x Samera ‘indigo’) ‘S304’ and violet-blue *P.* Purple Martin, whereas delphinidin was highly detected in blue-color *Delphinium grandiflorum* (Fig. 1h). These results suggest that all *Phalaenopsis* cultivars have cyanidin-based anthocyanin even though they have various colors, so we further examined factors affecting the violet-blue color of *Phalaenopsis* cultivars.

### Strong expression of *PeF3’H* was concomitant with cyanidin-based anthocyanin expression in various *Phalaenopsis* spp.

**Fig. 2:**
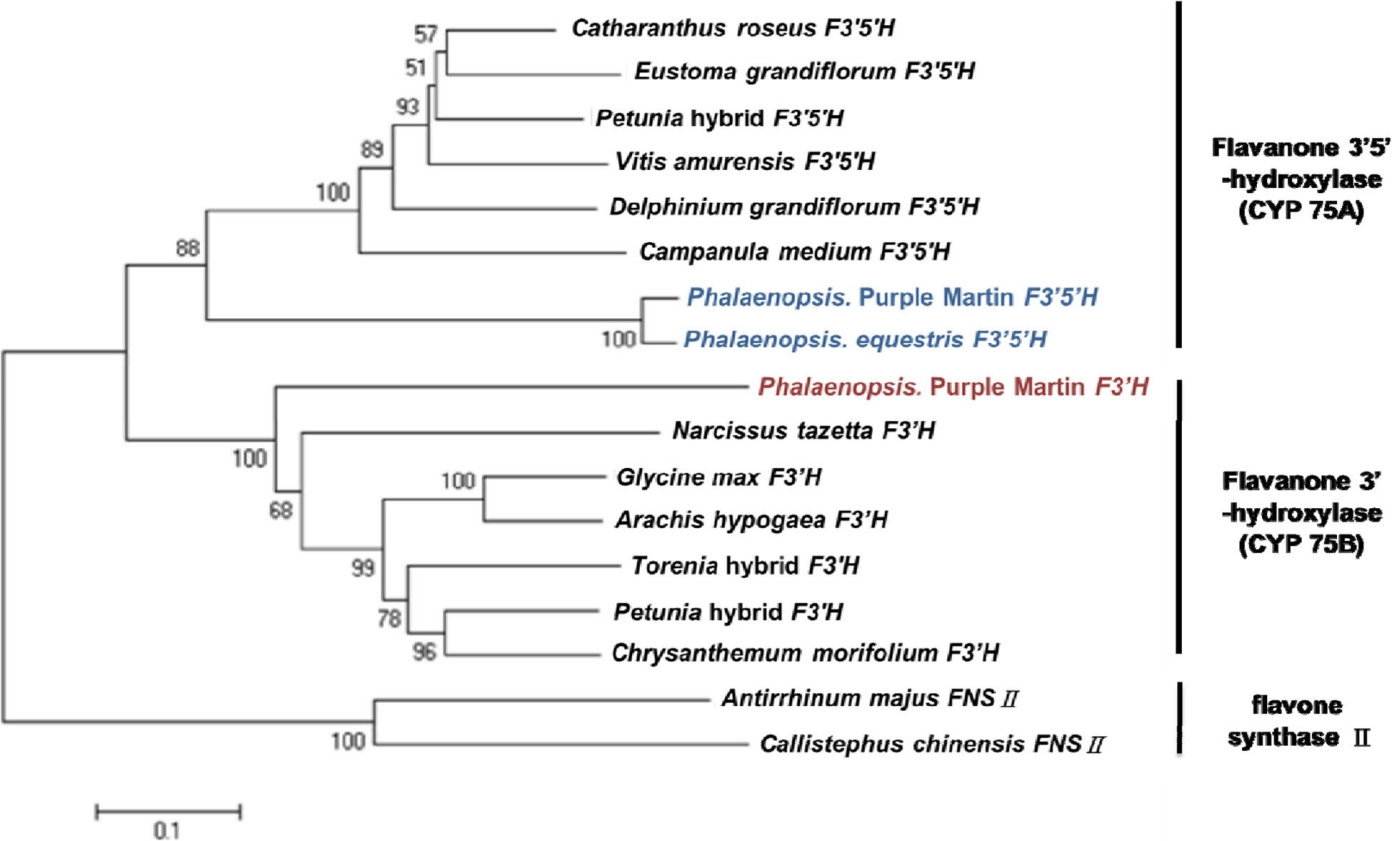
Phylogenetic tree of F3’5’H and F3’H. *F3’H from P. purple martin belongs to the cytochrome 75B family, F3’5’H from P. purple martin and Delphinium grandiflorum both belong to the cytochrome 75A family. Accession numbers of other species: Campanula medium F3’5’H (D14590), Catharanthus roseus F3’5’H (AJ011862), Eustoma grandiflorum F3’5’H (D14589), Petunia hybrida F3’5’H (Z22544), D. grandiflorum F3’5’H (AY856345), Vitis amurensis F3’5’H (FJ645756.1), Petunia hybrida F3’H (AF155332), Torenia hybrid cultivar F3’H (AB057673), Chrysanthemum morifolium F3’H (AB523844.1), Glycine max F3’H (AB191404), Narcissus tazetta F3’H (JX292106.1), Arachis hypogaea F3’H (JN572892.1), Antirrhinum majus FNSII (AB028151), and Callistephus chinensis FNSII (AF188612)*

**Fig. 3:**
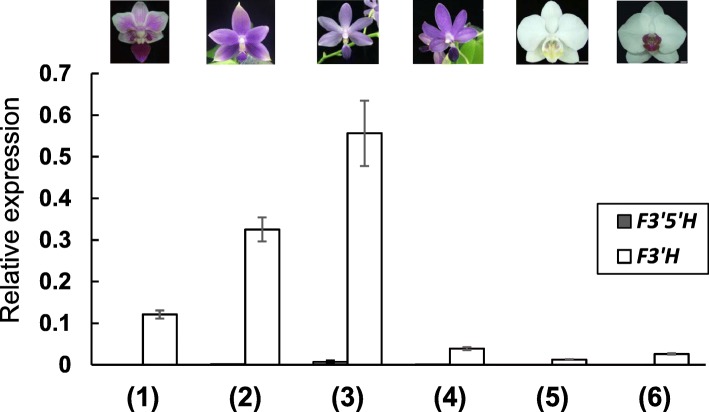
qRT-PCR analysis of *F3’H* and *F3’5’H*. *Expression of F3’H (white bar), and F3’5’H (gray bar) in various-colored Phalaenopsis cultivars including (1) P. hybrid ‘King Car’ (2) P. (Germaine Vincent x Samera ‘Indigo’) ‘S304’, (3) P. Kenneth Schubert, (4) P. Purple Martin, (5) P. aphrodite, and (6) P. OX Brother Seamate ‘OX1313’*

To study the differential gene expression of *F3’H* and *F3’5’H* in *Phalaenopsis*, *PeF3’H* was isolated from OrchidBase [23, 24] and *PhF3’5’H* was amplified from purple-color *P*. Purple Martin by using 5′- and 3′-RACE. We performed phylogenetic analysis with *PeF3’H* and *PhF3’5’H* and 12 flavanone hydroxylases from other plants (Fig. 2). *PeF3’H* clustered with the cytochrome 75B group and *PhF3’5’H* with the cytochrome 75A group. The expression of *PeF3’H* and *PhF3’5’H* in sepals and petals of floral buds from among various *Phalaenopsis* cultivars was examined by quantitative RT-PCR (qRT-PCR) (Fig. 3). *PeF3’H* level was higher than *PhF3’5’H* level in red-purple and purple cultivars and also violet and violet-blue cultivars. In contrast, *PeF3’H* and *PhF3’5’H* levels were low in white-color *P. aphrodite* and white-petals/red-lip *P.* OX Brother Seamate ‘OX1313’ (Fig. 3). This result indicates an association between the strong expression of *PeF3’H* and high accumulation of cyanidin-based anthocyanin in all *Phalaenopsis* cultivars.

### Accumulation of delphinidin-based anthocyanin and blue pigmentation formation resulted from ectopic overexpression of both *DgF3’5’H* and *PeMYB2*

**Fig. 4:**
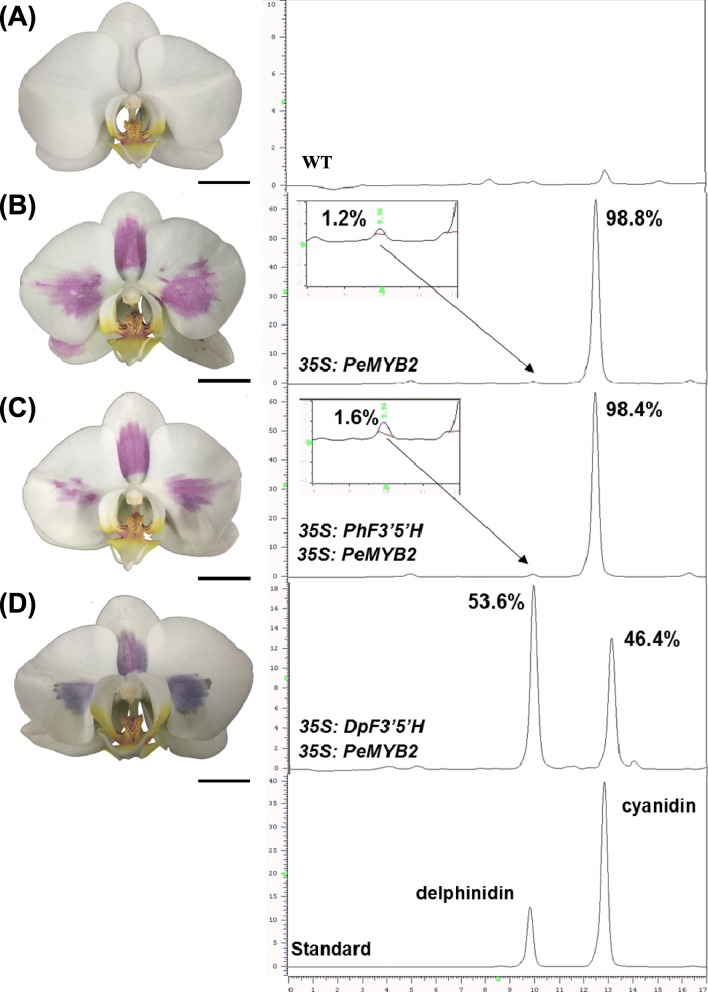
HPLC of anthocyanin compound in V3 with transient overexpression both PhF3’5’H or DgF3’5’H and PeMYB2. Transient overexpression of (**a**) wild-type (WT), (**b**) PeMYB2, (**c**) PhF3’5’H and PeMYB2, and (**d**) DgF3’5’H and PeMYB2 in V3. The left panel is the phenotype of flowers with or without 5-day A. tumefaciens infiltration and the right panel is HPLC analysis of anthocyanin compounds

To assess whether delphinidin could be accumulated in *Phalaenopsis* orchids, we used ectopic overexpression of *F3’5’H*. First, we confirmed whether PhF3’5’H from *Phalaenopsis* orchids has any enzyme activities. *Agrobacterium tumefaciens* containing *PhF3’5’H* was infiltrated into white tepals of *P*. Sogo Yukidian ‘V3’ (abbreviated as V3 hereafter) (Fig. 4a). We previously showed that transient overexpression of *PeMYB2* could increase the expression of downstream structural genes of *PeF3H*, *PeDFR* and *PeANS* and resulted in anthocyanin accumulation [22]. Overexpression of *PeMYB2* in white-color V3 resulted in 1.2% delphinidin and 98.8% cyanidin accumulation with a red-purple color (Fig. 4b). Overexpression of *PhF3’5’H* and *PeMYB2* resulted in 1.6% delphinidin and 98.4% red-purple cyanidin, which suggests that *PhF3’5’H* has no enzyme activity and no delphinidin accumulation resulted (Fig. 4c)*.* In contrast, overexpression of both *DgF3’5’H* (from *D. grandiflorum*) and *PeMYB2* resulted in 53.6% delphinidin and 46.4% cyanidin accumulation (Fig. 4d). These results suggest that a blue flower can result from functional *F3’5’H* present in *Phalaenopsis* and the activation of *F3H*, *DFR*, and *ANS* by PeMYB2.

### Substrate recognition site 6 (SRS6) of PeF3’H and PhF3’5’H and prediction of substrate docking

There are six substrate recognition sites (SRSs) near the heme group of F3’5’H; mutation of the individual amino acids at these sites might influence enzyme function [25–27].

**Fig. 5:**
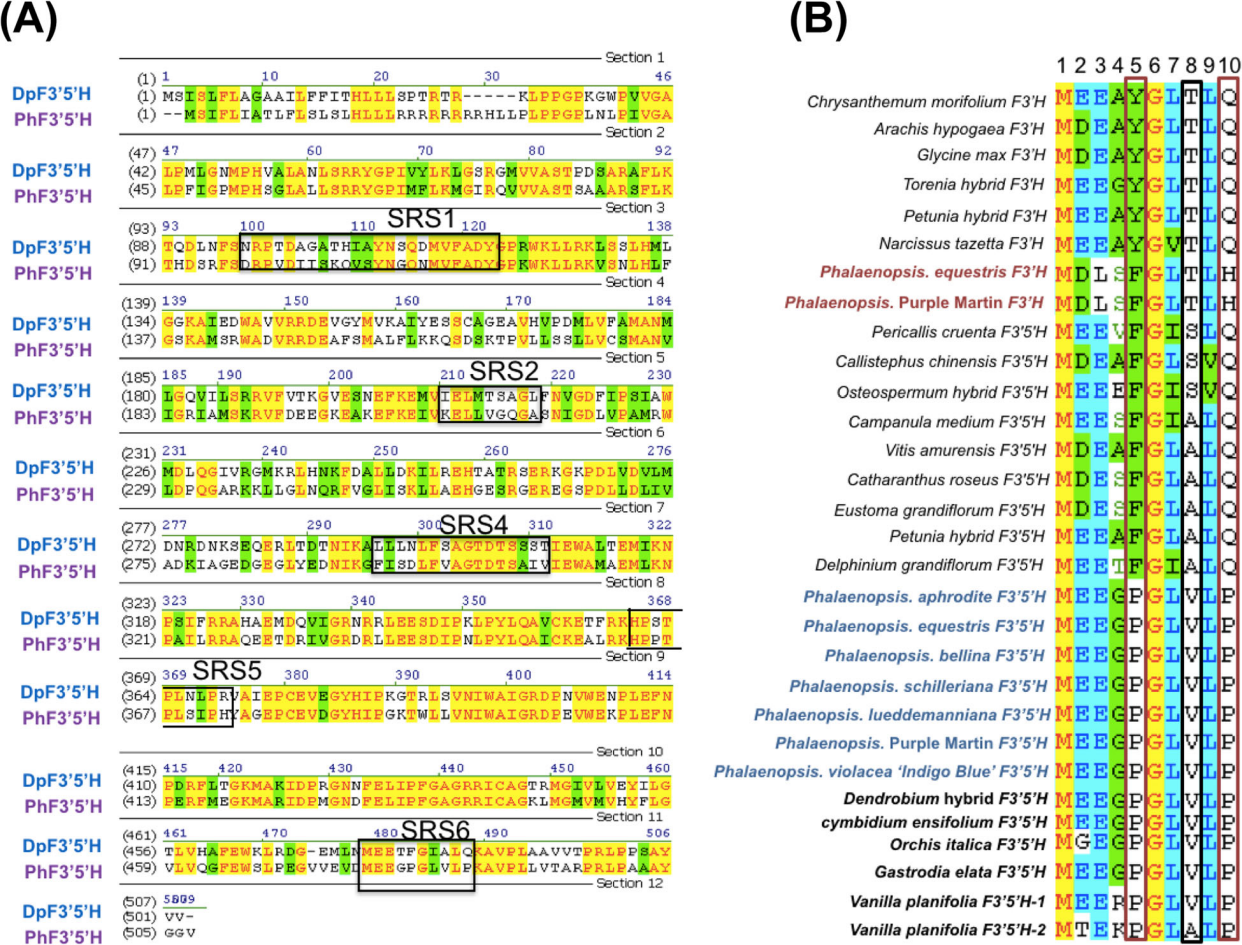
Multiple alignment of substrate recognition site 6 (SRS6) with F3’Hs and F3’5’Hs. **a** SRS1, SRS2, SRS4, SRS5 and SRS6 are boxed. **b** Multiple alignment analysis of F3’Hs and F3’5’Hs from Phalaenopsis (highlighted in red and blue, respectively) and other members of Orchidaceae including Dendrobium, Cymbidium, Orchis, Gastrodia and Vanilla (highlight in deep dark). Positions 5 and 10 of SRS6 amino acids are shown in red squares, and position 8 of SRS6 is shown in a black box. Accession numbers of other plants: *Campanula medium* F3’5’H (D14590), *Catharanthus roseus* F3’5’H (AJ011862), Pericallis cruenta F3’5’H (DQ257626.1), *Callistephus chinensis* F3’5’H (AAG49299.1), Osteospermum hybrid F3’5’H (DQ257627.1), *Eustoma grandiflorum* F3’5’H (D14589), *Petunia hybrida* F3’5’H (Z22544), *Delphinium grandiflorum* F3’5’H (AY856345), *Vitis amurensis* F3’5’H (FJ645756.1), *Petunia hybrida* F3’H (AF155332), Torenia hybrid cultivar F3’H (AB057673), *Chrysanthemum morifolium* F3’H (AB523844.1), *Glycine max* F3’H (AB191404), *Narcissus tazetta* F3’H (JX292106.1), *Arachis hypogaea* F3’H (JN572892.1)

To examine why *PhF3’5’H* cannot produce delphinidin, we wondered about divergence in amino acids in its sequences. The deduced amino acid sequences of *PhF3’5’H* were analyzed. They contained three conserved regions including an AGTDTS cytochrome P450 conversed region, EXXR oxygen binding motif and FGAGRRICAG heme binding domain (Fig. 5a). The amino acid sequences of SRS1, SRS2, SRS4, SRS5 and SRS6 domains in PhF3’5’H and DgF3’5’H are in Fig. 5a.

M [D/E] Ex [F/Y] Gx [T/S/A] xQ is the conserved region of SRS6, and position 8 of SRS6 is the crucial amino acid that determines functional divergence between F3’H and F3’5’H [26, 27]. Both PeF3’Hs of *Phalaenopsis* orchids have a threonine (T) residue at position 8 of SRS6, isolated from *P*. Purple Martin and *P*. hybrid ‘King Car’ (Fig. 5b). In contrast, all F3’5’Hs of *Phalaenopsis* orchids have a valine (V) residue at position 8 of SRS6, isolated from *P. aphrodite, P. equestris, P. bellina, P. schilleriana, P. lueddemanniana, P*. Purple Martin and *P. violacea* ‘Indigo Blue’ (Fig. 5b)*.* In addition, F3’5’Hs of other genera in Orchidaceae also have a V at position 8 of SRS6, including *Dendrobium* hybrid, *Cymbidium ensifolium*, *Orchis italic*, *Gastrodia elata* and *Vanilla planifolia* (Fig. 5b). One allele of *F3’5’H* from *V. planifolia* has an alanine (A) at position 8 of SRS6 (Fig. 5b). In addition, F3’5’Hs have two prolines (P) at positions 5 and 10 of SRS6 in Orchidaceae (Fig. 5b). In contrast, both phenylalanine (F) and glutamine (Q) are at positions 5 and 10 of SRS6 for *D. grandiflorum*.

**Fig. 6:**
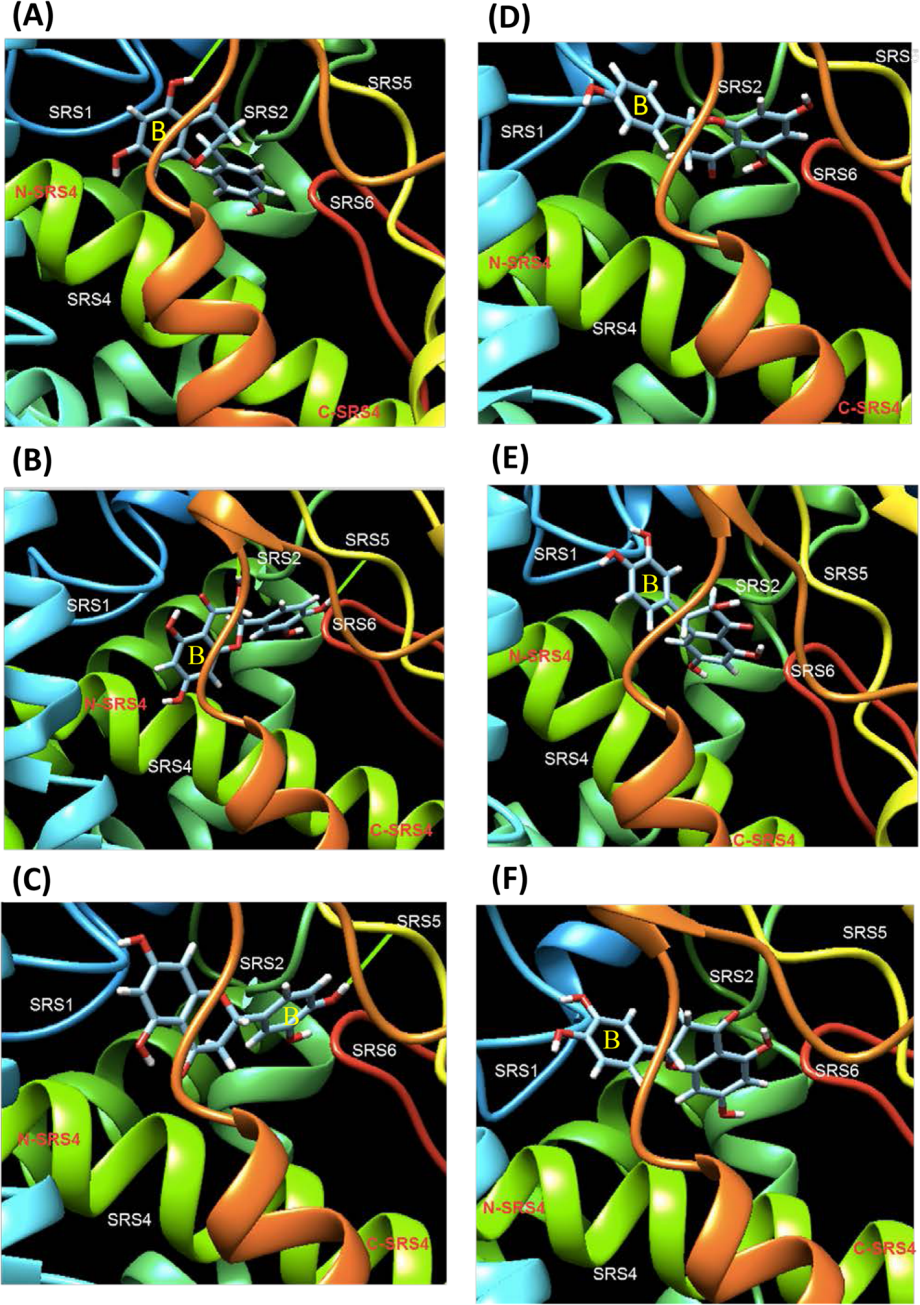
Substrate docking of F3’5’H and its substrates. *Substrate docking prediction of DgF3’5’H (****a*** *~* ***c****) and PhF3’5’H (****d*** *~* ***f****). The substrates used for docking are naringenin (****a****and****d****), dihydroquercetin (****b****and****e****), and eriodictyol (****c****and****f****). The B-ring is labeled as “B” (****a****-****f****)*

The substrate binding sites for N, DHQ and eriodictyol (E) on PhF3’5’H and DgF3’5’H were predicted by using SwissDock. Of the approximately 30 different conformations obtained in the substrate docking prediction with N, DHQ and E, the lowest energy conformation was selected as the most probable binding model. Prediction results showed that the aromatic rings (B-ring) of N, DHQ and E were oriented toward the N-terminal of SRS4 and SRS1 of PhF3’5’H (Fig. 6d-f). In contrast, the aromatic rings (B-ring) of N, DHQ and E were oriented toward the C-terminal of SRS4, SRS5 and SRS6 of DgF3’5’H (Fig. 6a-c).

### Violet-blue cultivars have higher vacuolar pH than do purple and white cultivars

**Fig. 7:**
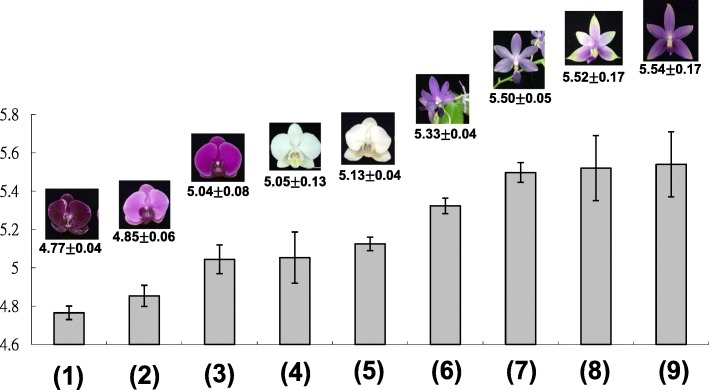
pH value in petal crude extracts of various-colored cultivars of *Phalaenopsis*. (1) *P*. OX Firebird ‘OX1506 mutant’, (2) *P*. OX Honey ‘OX1372’, (3) *P*. Big Chili, (4) *P*. Sogo Yukidian ‘V3’, (5) *P. aphrodite* subsp. *formosana*, (6) *P*. Purple Martin, (7) *P*. Kenneth Schubert, (8) *P. violacea* ‘Indigo blue’, and (9) *P. violacea* ‘KS1266’

To understand any divergent H^+^ concentrations among various-colored *Phalaenopsis*, the pH value was measured from flower extracts (Fig. 7). The pH values showed an increasing trend as *Phalaenopsis* flower colors became more violet blue (Fig. 7). *Phalaenopsis* cultivars with violet and violet-blue flowers had higher pH values than those with red-purple, purple and white flowers. Cultivars in the purple group, *P*. OX Firebird ‘OX1506 mutant’ and *P*. OX Honey ‘OX1372’, had pH values of 4.77 and 4.85, respectively (Fig. 7). The pH value of purple-violet *P*. Big Chili was 5.04 (Fig. 7). Cultivars in the violet group, *P. violacea ‘*Indigo blue’ and *P.* (Kenneth Schubert x Samera) ‘KS1226’, had pH values of 5.52 and 5.54, respectively (Fig. 7). Cultivars in the violet-blue group, *P.* Purple Martin and *P*. Kenneth Schubert, had pH values of 5.33 and 5.50, respectively (Fig. 7). Cultivars in the white group, *P. aphrodite* and V3, had pH values of 5.05 and 5.13, respectively (Fig. 7). These results suggest that decreased acidification enables the presentation of blue hues of *Phalaenopsis* orchids.

### Absorption spectra of anthocyanin extracts from *Phalaenopsis* orchids

**Fig. 8:**
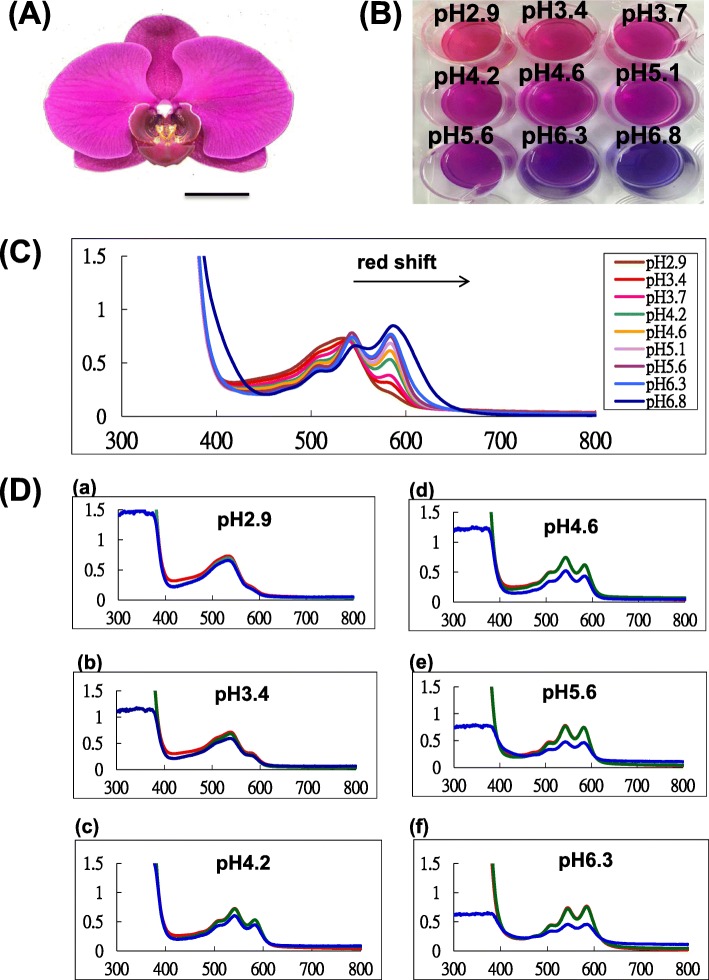
Absorption spectra of anthocyanin extract from *P.* Big Chili. *(A) Flower of P. Big Chili, (B) anthocyanin extract from flowers of P. Big Chili at different pH values, (D) absorption spectra of tepal crude extracts at different pH values. Scale bar is 2.5 cm. (D) Absorption spectra of anthocyanin extract at different times. Visible absorption spectra of tepal crude extracts from P. OX Honey ‘OX1372’ at various pH values, including (a) 2.9, (b) 3.4, (c) 4.2, (d) 4.6, (e) 5.6, and (f) 6.3 incubated for 0 h (red line), 24 h (green line) and 96 h (red line)*

To confirm whether anthocyanins under various levels of acidification will present different colors, anthocyanins were extracted from purple-violet *P.* Big Chili (Fig. 8a) and added into buffers adjusted to various pH values from 2.9 to 6.8 (Fig. 8b). The anthocyanins shifted toward a red hue in the acidic conditions and blue hue in basic conditions (Fig. 8b). In the absorption spectra of visible light, the red-shift of the wavelength occurred under basic conditions (Fig. 8c). In addition, anthocyanins were easily degraded in the alkaline solution (Fig. 8d). This result suggests that *Phalaenopsis* anthocyanins present a blue color in basic environments and are unstable in alkaline conditions with high pH values (Fig. 8d). In fact, the flower color faded easily among consecutive flowers in the same inflorescence of violet-blue *Phalaenopsis* cultivars (Additional file 2). The newly bloomed flower was purple, and it became a light purple color as the flowers faded (Additional file 2). The correlation between pH value and violet blue color formation was highly positive, as analyzed by Pearson’s correlation coefficient (R^2^ = 0.895).

### Positive association between increased concentration of metal ions and violet-blue color formation

We then compared the ratio of metal ions to anthocyanin between purple and violet-blue *Phalaenopsis* cultivars. Five metal ions including Mg^2+^, Al^3+^, Ca^2+^, Fe^3+^ and Zn^2+^ were analyzed in the purple color cultivar *P*. OX Honey ‘OX1372’, violet-blue color cultivar *P*. Kenneth Schubert, and dark-purple colored *P*. OX Firebird ‘OX1506 mutant’ by using ICP-MS (Table 1). The concentrations of cyanidin were 0.68, 0.26, and 10.74 μmol in purple *Phal.* OX Honey ‘OX1372’, violet-blue *P*. Kenneth Schubert, and dark-purple *P*. OX Firebird ‘OX1506 mutant’, respectively.

**Table 1:**
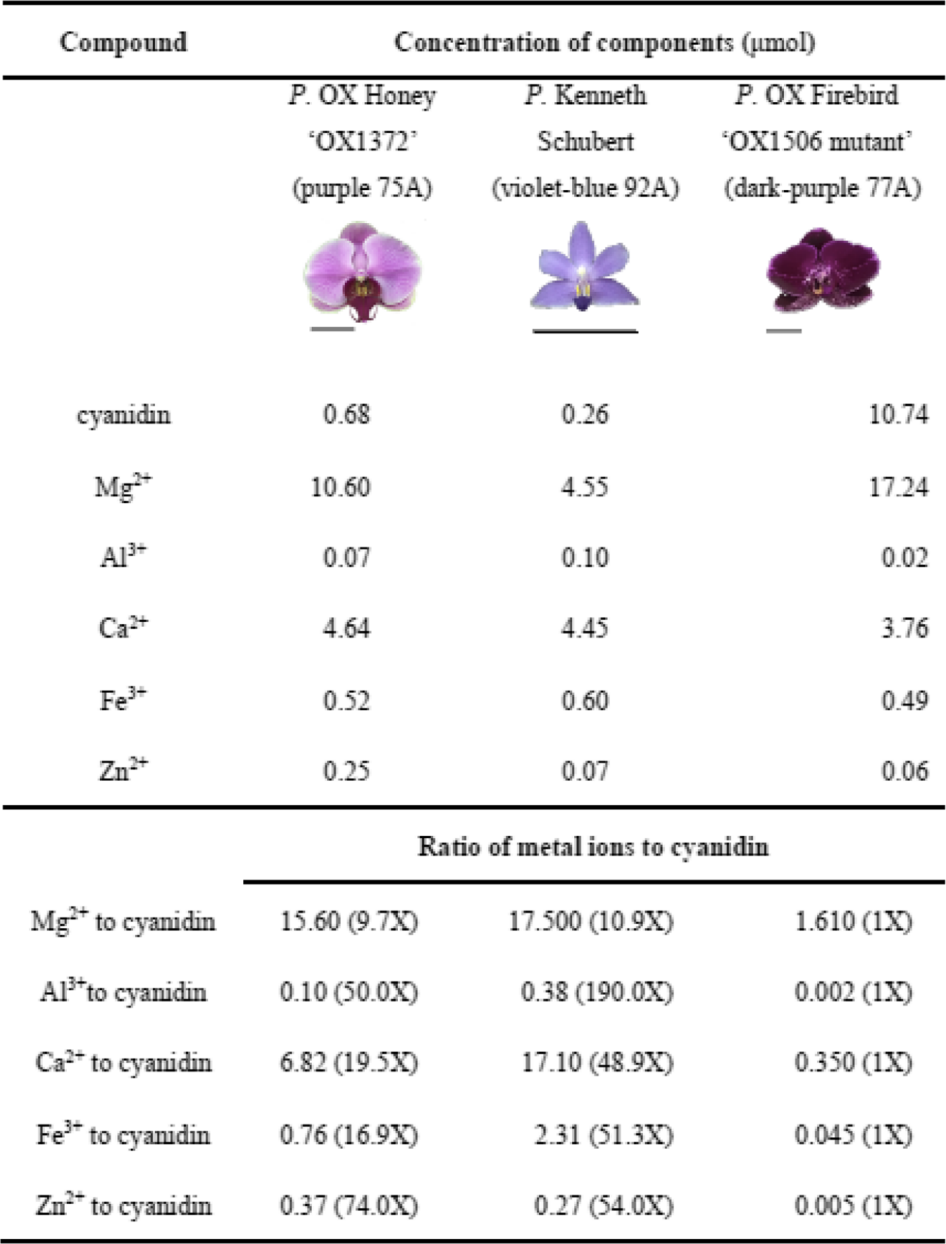
Concentrations of metal elements and anthocyanin and their molar ratio

The molar ratios of Al^3+^, Ca^2+^ and Fe^3+^ to cyanidin were 190-, 49-, and 51-fold higher, respectively, in violet-blue *P*. Kenneth Schubert than in dark-purple *P*. OX Firebird ‘OX1506 mutant’ (Table 1). In contrast, the molar ratios of Mg^2+^ and Zn^2+^ to cyanidin did not differ between violet-blue *P*. Kenneth Schubert and purple *P.* OX Honey ‘OX1372’. These results indicate divergence in molar ratios of Al^3+^, Ca^2+^ and Fe^3+^ to cyanidin-based anthocyanin between purple and violet-blue *Phalaenopsis*. Association between the molar ratios of metal ion to cyanidin and violet blue color formation was analyzed statistically by Pearson’s correlation coefficient. The positive correlation between the molar ratios of Al^3+^, Ca^2+^ and Fe^3+^ to cyanidin and violet blue color formation was high (R^2^ = 0.94, 0.88 and 0.91, respectively), whereas that between the molar ratios of Mg^2+^ and Zn^2+^ to cyanidin and blue color formation was low (R^2^ = 0.5 and 0.15, respectively).

## Discussion

### Same anthocyanin compound but different vacuolar pH values between violet-blue and purple-red flowers

Vacuolar pH is higher in blue flowers than red flowers in petunia, morning glory and hydrangea, but blue and red flowers have the same anthocyanin compound [10, 14, 28]. This indicates that vacuolar pH is a significant factor regulating flower color among red to blue colors when observing different colored flowers with the same anthocyanin compound accumulated. In *Phalaenopsis* orchids, cyanidin-based anthocyanin was highly accumulated in flowers from red-purple to violet-blue. However, violet-blue *Phalaenopsis* cultivars have higher pH in flower sap than do purple cultivars.

### Color-shift from violet-blue to purple might be regulated by the divergence of molar ratio of metal ions to cyanidin-based anthocyanin in *Phalaenopsis*

Previous studies showed that blue-red bicolor tulips had a molar ratio of Fe^3+^ to delphinidin-based anthocyanin 39-fold higher in blue than red cells [29]. In blue and red hydrangea flowers, the molar ratio of Al^3+^ to delphinidin-based anthocyanin was 40-fold higher in blue than red flowers [30]. In blue and purple cornflowers, the molar ratio of Fe^3+^ to cyanidin-based anthocyanin was 51-fold higher in blue than purple flowers [31]. Fe^3+^ is necessary to form the blue color in cyanidin**-**based flowers including cornflower and blue poppy [17, 32, 33]. Mg^2+^ is essential for producing metalloanthocyanin in several plants, including *Commelina communis*, *Centaurea cyanus*, *Salvia patens*, *S. uliginosa* and *Nemophila menziesli* [16]. Our results showed that the molar ratio of Al^3+^, Ca^2+^ and Fe^3+^ metal elements to cyanidin-based anthocyanin was 190-, 49-, and 51-fold higher, respectively, in violet-blue than purple *Phalaenopsis*.

### Interference in protein functions by the substitution of amino acids in SRS domains and substrate orientation

The large size of amino acids results in eliminating F3’5’H function. Substitution of Ser to Thr at position 8 of SRS6 can decrease 5′-hydroxylation activity [26]. Similarly, sequence analysis showed Val in PhF3’5’H and Ala in DgF3’5’H, correspondingly, which may indicate why PhF3’5’H lost the 5′-hydroxylation activity. In addition, replacements of amino acid residues of the SRS4 domain in F3’5’H of soybean (GmaxF3’5’H) could also alter its enzymatic activityby disrupting the contact between heme group and substrate [34]. This affects the conformation of the active site in F3’5’H and thus change the flower color formation. As compared with the amino acid residues of the SRS4 domain of GmaxF3’5’H, there are more amino acid residues altered in PhF3’5’H than DgF3’5’H [34]. The amino acid sequence of the SRS4 domain in DgF3’5’H is similar to that of GmaxF3’5’H, except Ser300 replaces Thr300. The side-chain of residue 300 points away from that heme; thus, the substitution of this residue does not eliminate the function. Even more amino acid residues are altered in PhF3’5’H. Most side chains of these substitutes in the model structure are located away from the heme, except for Phe294 and Asp297, which are located near the heme; thus, most substitutions do not alter the function. Phe294Leu and Asp297Asn may interfere in the protein function by altering the conformation of active sites via a large size and charge repulsion, respectively.

In addition, results of substrate docking showed that the aromatic rings (B-ring) of N, DHQ and E were oriented toward the N-terminal of SRS4 and SRS1 of PhF3’5’H (Fig. 6), whereas the aromatic rings (B-ring) of N, DHQ and E were oriented toward the C-terminal of SRS4, SRS5 and SRS6 of DgF3’5’H. This finding is similar to previous results in predicting F3’H in *Arabidopsis thaliana* [35]. PhF3’5’H may fail to function because of its unusual direction of substrate binding. Thus, homology modeling and docking can provide a good model to evaluate experimentally tested mutants and as a further rationale for the effect of individual mutants.

### True blue *Phalaenopsis* flowers could be achieved by genetic engineering of the anthocyanin biosynthesis pathway

Endogenous *F3’H* must be silenced for high delphinidin accumulation to lead to a blue hue because both *F3’H* and *F3’5’H* compete for the same substrates of N and DHK [8]. For transgenic chrysanthemum, 80 and 35% delphinidin accumulated in *F3’5’H*-induced plants with and without endogenous *F3’H* silenced [8]. Previous studies showed that 80 and 95% delphinidin accumulated and resulted in purple-violet (N81B) and violet (88C) chrysanthemum [9]. In addition, by overexpressing both heterologous *F3’5’H* and *PhDFR*, 94 and 100% delphinidin accumulation resulted in purple 82A and mauve 75A groups, respectively, in carnation [6, 7]. In rose, overexpression of heterologous *F3’5’H* and *PhDFR* resulted in a 95% delphinidin accumulation and the violet group 85B [5]. In our study, overexpression of both *PeMYB2* and *DgF3’5’H* in the V3 tepal caused the highest delphinidin accumulation (53.6%), and the flower color shifted from white (NN155C) to violet-blue (91A). Even though relatively lower delphinidin accumulation was observed with overexpression of *DgF3’5’H* in *Phalaenopsis*, higher delphinidin accumulation and a bluer violet-blue resulted as compared with other overexpressed plants. This is the first report to show a novel blue hue created in white *Phalaenopsis* cultivars. Our findings suggest that with the addition of PeMYB2, DgF3’5’H was able to compete the substrates of N and DHK with endogenous *F3’H* in *Phalaenopsis* to accumulate the delphinidin compound. Silencing endogenous *F3’H* accompanied by *DgF3’5’H*-induced transgenic *Phalaenopsi*s is required to produce high delphinidin accumulation and a true-blue color formation in *Phalaenopsis*.

Different strategies of genetic regulation in the anthocyanin biosynthesis pathway have been used [36]. Heterologous *F3’5’H* and also *PhDFR* were introduced into host plants of rose and carnation. *PhDFR* strongly utilizes dihydromyricetin (DHM) but not DHK, thereby benefiting delphinidin production [5–7]. In contrast, to produce blue chrysanthemum, heterologous *F3’5’H* was introduced, and endogenous *F3’H* was silenced. Knockdown of *F3’H* can increase delphinidin production [8]. Of note, for the strategy adopted in chrysanthemum, only the suitable promoter of chrysanthemum F3H was used to drive efficient heterologous expression of F3’5’H without modifying other structure genes in the anthocyanin biosynthesis pathway and led to a high production of delphinidin and accumulation in transgenic chrysanthemum [9]. These cases were all successful in causing a high production of at least 80% delphinidin by regulating the anthocyanin biosynthesis pathway and leading to a novel blue hue.

In conclusion, we proved the initial assumption framework of how white-color orchids are converted to blue hue by overexpressing an anthocyanin regulating *MYB* transcription factor, *PeMYB2*, and heterologous *F3’5’H* (*DgF3’5’H*) while knocking down the expression of endogenous *F3’H*. This can set the ground work for molecular breeding of *Phalaenopsis* cultivars with real blue pigment.

## Conclusions

The violet-blue color formation in *Phalaenopsis* was caused by both reduced vacuolar acidification and enhanced molar ratio between Ca^2+^ and Fe^3+^ to cyanidin-based anthocyanin. Delphinidin did not accumulate in any colors of *Phalaenopsis* cultivars because of the altered amino acids and possibly adverse substrate binding direction, *PhF3’5’H* has little to no ability for delphinidin accumulation. On overexpressing both *DgF3’5’H* and *PeMYB2*, we observed a novel violet-blue color in the white *Phalaenopsis* cultivar, with 53.6% delphinidin and 46.3% cyanidin accumulation*.* This study will benefit the understanding of the regulatory mechanism of violet-blue color formation and set the groundwork for molecular breeding of *Phalaenopsis* cultivars with novel blue color pigmentation.

## Methods

### Plant materials

For color analysis, *Phalaenopsis* cultivars with various colors including red-purple, purple, purple-violet, violet, violet-blue and white were collected from various local orchid nurseries (Additional file 1). For comparison, blue *Delphinium grandiflorum* was collected from Ruifeng Horticulture (Changhua, Taiwan) (Additional file 2). Among the *Phalaenopsis* cultivars, *P*. OX Brother Seamate ‘OX1313’ with white sepals/petals and red-purple lip, purple *P*. OX Honey ‘OX1372’ and dark-purple *P*. OX Firebird ‘OX1506 mutant’ were collected from OX Orchids Farm (Tainan, Taiwan). Purple *P.* hybrid ‘King Car’ was collected from King Car Biotechnology Industrial Co. (Ilan, Taiwan). Purple-violet *P*. Big Chili, white *P. aphrodite* subsp. *formosana*, and white *P*. Sogo Yukidian ‘V3’ were collected from Taiwan Sugar Corp. (Tainan, Taiwan). Violet *P. violacea* ‘Indigo Blue’ was collected from Meidarland Orchids (Tainan, Taiwan). Violet *P.* (Germaine Vincent x Samera ‘indigo’) ‘S304’ was collected from Keng-Liang Liu Orchid Nursery (Kaohsiung, Taiwan). Violet *P.* (Kenneth Schubert x Samera) ‘KS1226’ and Violet-blue P. Purple Martin were collected from Kung Sir Orchids (Tainan, Taiwan). Violet-blue *P. tau* Chiang Sapphire was collected from Sunhope Garden Biotec (Tainan, Taiwan). Violet-blue *P.* Kenneth Schubert was collected from Taida Horticultural Co. (Changhua, Taiwan). The white *P*. Sogo Yukidian ‘V3’ and purple *P*. OX Honey ‘OX1372’ were used for transient overexpression analysis because their colors of tepals were beneficial for delphinidin accumulation and blue color. All plant materials were collected according to institutional guidelines and grown in the greenhouse at Nation Cheng Kung University (NCK, Tainan, Taiwan) under natural light and at 23–27 °C. All plant materials used in this study including the *Phalaenopsis* cultivars and *Delphinium grandiflorum* are commercially available, not collected from the wild. All plant materials were kept in permissible green house at NCKU. Experimental research on plants comply with institutional, national, or international guidelines. The authenticity of these materials has been verified by each orchid nursery owners.

### Definition of flower colors

For the red-purple series of plant materials, their colors ranged from red-purple, purple, purple-violet, violet and violet-blue in *Phalaenopsis* (Additional file 1). To define the colors, the Royal Horticultural Society Color Chart was used. Lip color of *P*. OX Brother Seamate ‘OX1313’ is classified in the red-purple 71A group (Additional file 2). *P*. OX Honey ‘OX1372’, *P*. OX Firebird ‘OX1506 mutant’ and *P*. hybrid ‘King Car’ are classified in the purple 75A, 77A and N78C groups, respectively (Additional file 2). *P*. Big Chili is classified in the purple-violet N80A group (Additional file 2). *P. violacea ‘*Indigo blue’, *P.* (Germaine Vincent x Samera ‘indigo’) ‘S304’ and *P.* (Kenneth Schubert x Samera) ‘KS1226’ are classified in the violet 86A, N87A and N87B groups, respectively (Additional file 2). *P. tau* Chiang Sapphire, *P.* Purple Martin and *P*. Kenneth Schubert are classified in the violet-blue 90A, 90B and 92A groups, respectively (Additional file 2). *P. aphrodite* subsp. *formosana* and *P*. Sogo Yukidain ‘V3’ are both classified in the white NN155C group (Additional file 2). For comparison, *Delphinium grandiflorum* is classified in the blue 101A group (Additional file 2).

### Extraction of anthocyanin

Four days post transient overexpression in V3 flowers, anthocyanins were extracted as previously described and quantified with HPLC approach [37]. Briefly, the ground sample powders were extracted with methanol supplied with 1% (v/v) HCl at 4 °C for 20 h. The samples were then centrifuged at 10,000 Xg for 20 min at 4 °C. The supernatant was dried using a vacuum concentrator, and the pellet was resolved in 2 N HCl and hydrolyzed the glycosyl group at 100 °C for 1 h. The hydrolyzed samples were retrieved through the solid-phase DSC-18 SPE extraction column (Supelco, SL, USA) and then eluted in methanol supplied with 1% (v/v) HCl. The eluents were stored at − 20 °C for HPLC analysis. We used 250 × 4.6-mm Hypersil BDS C18 column (Thermo Fisher Scientific, MA, USA) for HPLC separation (Hitachi d-7000, l-7100, L7200, and L7420). Solvent A (formic acid: water = 1:99 [v/v]) and solvent C (100% methanol) were mixed at a flow rate of 1.0 ml/min for compound resolution. Anthocyanins were detected at the 530 nm wavelength. Cyanidin (Sigma-Aldrich, SL, USA) was recruited as a standard in HPLC analysis. Three biological repeats were performed for each overexpression experiments, and repeated transient assays twice independently.

### RNA extraction and reverse transcription to cDNA

Sepals and petals of 1- to 2.5-cm floral buds were collected, submerged in liquid nitrogen and then stored at − 80 °C for RNA extraction. Total RNA was extracted as described previously by using guanidium thiocyanate method [37]. Residual DNA was removed by treating with RNase-Free DNase I (New England Biolabs, MA, USA). Reverse transcription to cDNA was performed using SuperScript III (Invitrogen, CA, USA).

### qRT-PCR

For each gene, primer pairs were designed within the gene-specific regions (Additional file 3). q-PCR by amplification of cDNA with a fluorescent SYBR Green PCR Master Mix (Applied Biosystems, MA, USA) dye were used to measure gene expression and detected by an ABI Prism 7000 Sequence Detection System (Applied Biosystems, MA, USA). Reactions were performed at 95 °C for 10 min and repeated cycling for 40 cycles (95 °C for 15 s and 60 °C for 1 min). After magnification, melting curve analysis was used to verify primer dimer formation and amplicon specificity. *PeActin4* (AY134752), a housekeeping gene, was used for normalization of qRT-PCR results [38]. Each sample was analyzed in technical triplicate. Data are presented as mean ± SD of three technical replicates and three independently biological samples performed.

### Sequence alignment and phylogenetic analysis

Sequence alignment involved using Align X (Vector NTI Suite, V.8, Invitrogen, Carlsbad, CA, USA). Phylogenetic analysis trees were constructed with the neighbor-joining method and evaluated by bootstrap analysis with MEGA5 [39], with 1000 bootstrapping datasets to estimate the confidence of each tree clade. Two different phylogenetic trees were constructed: one was constructed with sequences of 7 *F3’Hs*, 7 *F3’5’Hs* and 2 *FLSIIs* and the other was constructed with sequences of 23 *ATPases.* Sequences of *F3’Hs*, *F3’5’Hs*, *FLSIIs,* and *ATPases* from other plants were acquired from GenBank (http://www.ncbi.nlm.nih.gov/), with the following accession numbers: *Campanula medium* F3’5’H (D14590), *Catharanthus roseus* F3’5’H (AJ011862), *Eustoma grandiflorum* F3’5’H (D14589), *Petunia hybrida* F3’5’H (Z22544), *Delphinium grandiflorum* F3’5’H (AY856345), *Vitis amurensis* F3’5’H (FJ645756.1), *Petunia hybrida* F3’H (AF155332), *Torenia hybrid cultivar* F3’H (AB057673), *Chrysanthemum morifolium* F3’H (AB523844.1), *Glycine max* F3’H (AB191404), *Narcissus tazetta* F3’H (JX292106.1), *Arachis hypogaea* F3’H (JN572892.1), *Antirrhinum majus* FNSII (AB028151), *Callistephus chinensis* FNSII (AF188612), *Arabidopsis thaliana* AtAHA2 (AEE85731.1), *Arabidopsis thaliana* AtAHA4 (Q9SU58.2), *Nicotiana plumbaginifolia* NPPMA1 (AAA34094), *Petunia x hybrida* PhPH5 (DQ334807), *Nicotiana plumbaginifolia* NpPMA9 (AF156684), *Populus trichocarpa* PtAHA10-like (XP_002326625.1), *Vitis vinifera* VvAHA10-like (CBI35782), *Oryza sativa* OsAHA9 (AJ440220), *Arabidopsis thaliana* AtAHA10 (NP173169), *Citrobacter sp.* CsMgtA (ZP04559661), *Escherichia coli* EcMgtA (YP672334), *Klebsiella pneumoniae* KpMgtA (YP002917472), *Petunia x hybrida* PhPH1 (AHH24342.1), *Vitis vinifera* VvPH1-like (CBI41039), *Ricinus communis* RcPH1-like (XP002533565), *Populus trichocarpa* PtPH1like (XP002306511), *Arabidopsis thaliana* AtACA2 (NP195497) and *Oryza sativa* Os03g0616400 (NP001050661).

### Molecular modeling and docking

The prediction of F3’5’H tertiary protein structures involved using SWISS-MODELL (https://swissmodel.expasy.org/). The X-ray crystal of P450 1A2 (PDB ID: 2hi4.1) was used as the template [40]. Next, the prediction of docking between the F3’5’H protein structure and substrates involved using SwissDock (http://www.swissdock.ch/docking). Finally, UCSF Chimera was used to visualize the predicted binding model [41].

### Transient overexpression of heterologous *F3’5’H*s and *PeMYB2* in *Phalaenopsis* flowers

For transient overexpression of *PeMYB2* and *PhF3’5’H* or *DgF3’5’*H in white-flower V3, the binary vector p1304NhXb containing duplicated Cauliflower mosaic virus (CaMV) 35S promoter was used following Hsu et al., (2015) [22]. Briefly, these genes were PCR-amplified and digested with restriction enzyme *Xho*I, and then ligated to p1304NhXb. The recombinant constructs were bombarded into *Agrobacterium tumefaciens* EHA105 using electroproration. The engineered *A. tumefaciens* was grown at 28 °C overnight, refreshed culture to OD_600_ = 0.8–1, and then harvested by centrifugation. Cell pellets were resuspended in Murashige and Skoog medium supplied with 100 μM acetosyringone, and incubated at room temperature for 30 min. The bacterial suspensions were infiltrated into the base of sepals and petals of V3 flowers, and incubated at 25 °C with a 10 h light/14 h dark photoperiod. Five days post bombardment, the infiltrated flowers were photographed and then stored at − 80 °C for anthocyanin extraction. The transient overexpression assay was performed in five different plants for each experiment, and repeated three times independently.

### pH value measurement and visible absorption spectra of corolla homogenates

The complete petals of flowers were directly ground, centrifuged at 13,000 rpm (Heraeus Biofuge Pico, Germany) for 15 min, then supernatant was immediately measured by using a pH electrode (Van London Co., TX, USA). In addition, the flower sap was diluted in 1 ml buffer solution containing 0.2 M sodium phosphate dibasic (Na_2_HPO_4_) and 0.1 M citric acid (C_6_H_8_O_7_) at various pH values and measured by spectrophotometric analysis from OD 300–800 nm (Biochrom Libra S50, UK).

### Metal ion measurement of the colorful petals

Powders of colorful petals were dried by using a freeze dryer (MILLROCK LYOBT85, USA), then ground by using a homogenizer (QIAGEN Schwingmuhle TissueLyser 2, Germany). Next, 100 mg of the dried sample was digested in 5 ml of 70% HNO_3_ and 0.5 ml 30% H_2_O_2_ for high-performance microwave treatment (CEM, MARS Xpress 5, Matthews, NC, USA) at 400 watts and increased to 180 °C within 15 min and sustained for 15 min. After digestion, samples were diluted with 2% HNO_3_ for metal ion measurement by using ICP-MS analysis (Agilent 7500cx, USA). High-performance microwave and ICP-MS were both performed at the Institute of Biomedical Engineering and Nanomedicine of National Health Research Institutes (Miaoli, Taiwan).

### Statistical analysis

The correlation between the concentration of metal ion and violet blue color formation and pH value and violet blue color formation were analyzed statistically with Pearson’s correlation coefficient [42].

## Supplementary information

**Additional file 1: **Flowers used in this study, and their definition of color code. *Flower color of Phalaenopsis and Delphinium hybrid were assigned according to the Royal Horticultural Society Color Chart.*

**Additional file 2: **Flower color of various flowering development stages of violet-blue *P*. Kenneth Schubert and *P*. Purple Martin.(A) *P.* Kenneth Schubert in various flowering development stages range from flower opening (D) and after flower opening 5 days (D + 5), 10 days (D + 10), 15 days (D + 15), and 20 days (D + 20). (B) Flowers of *P.* Kenneth Schubert and, (C) the whole plant of *P.* Purple Martin. Scale bar is 2.5 cm.

**Additional file 3: **Primers used in this study. *List of primers for qRT PCR, gene isolation, 5′ and 3′ RACE.*

## Data Availability

The datasets used and/or analysed during the current study available from the corresponding author on reasonable request.

## Abbreviations

ANS: Anthocyanin synthase
DFR: Dihydroflavonol-4-reductase
DHF: Dihydroflavonols
DHK: Dihydrokaempferol
DHQ: Dihydroquercetin
E: Eriodictyol
F3’5’H: Flavonoid 3′, 5′-hydroxylase
F3’H: Flavonoid 3′-hydroxylase
F3H: Flavonoid 3-hydroxylase
HPLC: High-performance liquid chromatography
ICP-MS: Inductively coupled plasma mass spectrometry
N: Naringenin
RACE: Rapid amplification of cDNA ends
RHSCC: Royal horticultural society color chart
SRS: Substrate recognition site
VIGS: Virus-induced gene silencing
V3: *Phalaenopsis* Sogo Yukidian ‘V3’
