# Supplementary material for: Assessment of violet-blue color formation in Phalaenopsis orchids
Source: BMC Plant Biol. 2020 May 12;20:212. doi: 10.1186/s12870-020-02402-7 (PMC7218627; doi:10.1186/s12870-020-02402-7)
Supplement: Supplementary file 1 — Additional file 1: Flowers used in this study, and their definition of color code. Flower color of Phalaenopsis and Delphinium hybrid were assigned according to the Royal Horticultural Society Color Chart. [file 12870_2020_2402_MOESM1_ESM.pdf]

	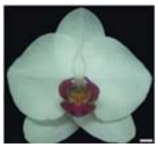	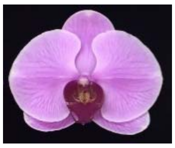	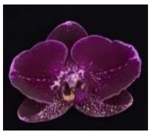	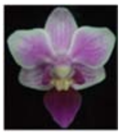	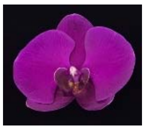
**Cultivar**	*P*. OX Brother Seamate 'OX1313'	*P*. OX Honey 'OX1372'	*P*. OX Firebird 'OX1506 mutant'	*P*. hybri*d* 'King Car'	*P*. Big Chili
**RHSCC**	red-purple 71A	purple 75A	purple 77A	purple N78C	purple-violet N80A

	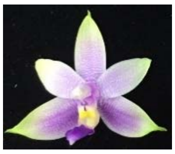	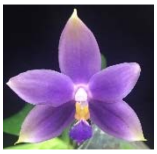	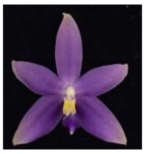	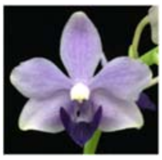	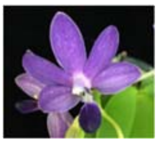
**Cultivar**	*P. violacea* 'Indigo blue'	*P.* (Germaine Vincen x Samera 'indigo') "S304"	*P.* (Kenneth Schubert x Samera) 'KS1226'	*P.* Tau Chiang Sapphire	*P.* Purple Martin
**RHSCC**	violet 86A	violet 87A	violet N87B	violet-blue 90A	violet-blue 90B

	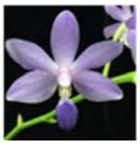	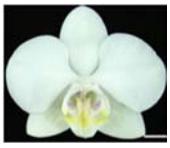	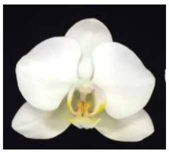	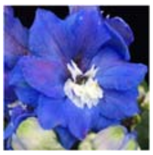
**Cultivar**	*P*. Kenneth Schubert	*P*. *aphrodite* subsp. *formosana*	*P*. Sogo Yukidian 'V3'	*Delphinium* hybrid
**RHSCC**	violet-blue 92A	white NN155C	white NN155C	blue 101A

Additional file 1. Flowers used in this study, and their definition of color code. Flower color of *Phalaenopsis* cultivars and *Delphinium* hybrid were assigned according to the RHSCC.
