# Supplementary material for: Assessment of violet-blue color formation in Phalaenopsis orchids
Source: BMC Plant Biol. 2020 May 12;20:212. doi: 10.1186/s12870-020-02402-7 (PMC7218627; doi:10.1186/s12870-020-02402-7)
Supplement: Supplementary file 2 — Additional file 2: Flower color of various flowering development stages of violet-blue P. Kenneth Schubert and P. Purple Martin.(A) P. Kenneth Schubert in various flowering development stages range from flower opening (D) and after flower opening 5 days (D + 5), 10 days (D + 10), 15 days (D + 15), and 20 days (D + 20). (B) Flowers of P. Kenneth Schubert and, (C) the whole plant of P. Purple Martin. Scale bar is 2.5 cm. [file 12870_2020_2402_MOESM2_ESM.pdf]

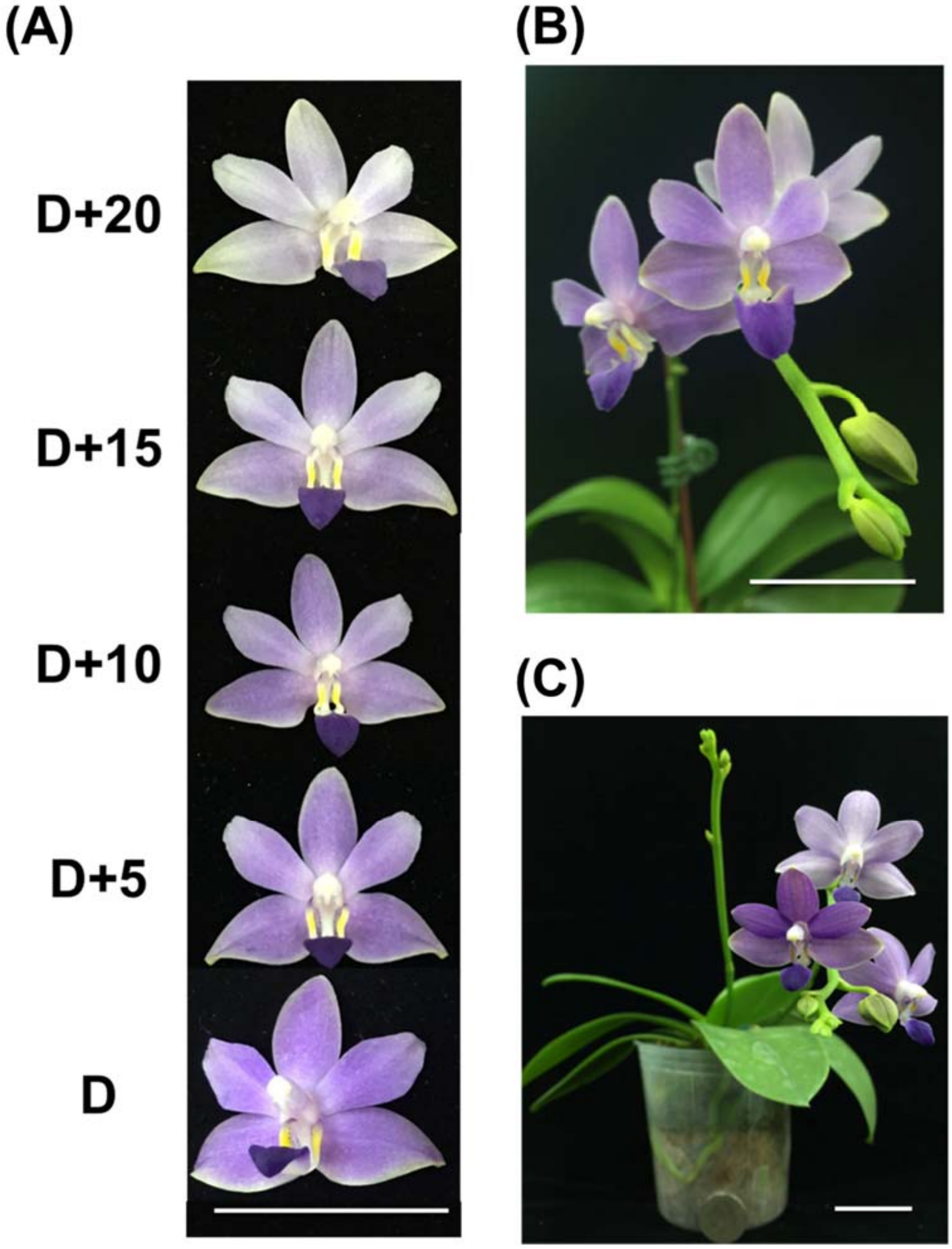

Additional file 2. Flower color of various flowering development stages of violet-blue *P*. Kenneth Schubert and *P*. Purple Martin.

(A) *P*. Kenneth Schubert in various flowering development stages range from flower opening (D) and after flower opening 5 days (D+5), 10 days (D+10), 15 days (D+15), and 20 days (D+20). (B) Flowers of *P*. Kenneth Schubert and, (C) the whole plant of *P*. Purple Martin. Scale bar is 2.5 cm.
