# Supplementary material for: Assessment of violet-blue color formation in Phalaenopsis orchids
Source: BMC Plant Biol. 2020 May 12;20:212. doi: 10.1186/s12870-020-02402-7 (PMC7218627; doi:10.1186/s12870-020-02402-7)
Supplement: Supplementary file 3 — Additional file 3: Primers used in this study. List of primers for qRT PCR, gene isolation, 5′ and 3′ RACE. [file 12870_2020_2402_MOESM3_ESM.pdf]

Additional file 3. Primers used in this study.

Primer name	5’→ 3’ nucleotide sequences
qRT PCR	
PhF3’5’H_QPCR_F2	TTGGCGGAGCACRGTGAGAGTAGAG
PhF3’5’H_QPCR_R2	CCGGCGATCTTATCAGCCACAA
PhF3’H_QPCR_F2	GACCACCAGATCGACGGCTACCTA
PhF3’H_QPCR_R2	GGGGAGGAACCKATCTGGCCG
PeAHA1_QPCR_F2	CCCTCTTCAGCGACAAGAGCAGC
PeAHA1_QPCR_R2	TGACTCCACATGTCCCTTCAATGTATTC
PeAHA2_QPCR_F1	CTCAGYGGAAAGGCATGGGACAAC
PeAHA2_QPCR_R1	CATTGAAGACTGATGTAGTCTCAGCG GTT
PeAHA3_QPCR_F1	ACAACTTATCACCWACATTTCAAATCT TGTTG
PeAHA3_QPCR_R1	AAAGCGCCGCGGTAAGCTGG
PeAHA4_QPCR_F1	TCGTGATACCGTCATCCTAATGGCG
PeAHA4_QPCR_R1	GATTGAAAGGCAAAAAATGAACTTCT TGGATAT
PeAHA5_QPCR_F2	CACCRCTACCTGATAAGTGGAACCTCA CC
PeAHA5_QPCR_R2	TCGGTCTGATGAGCTGCATAATAGAATA GA
Gene isolation	
PM_F3’5’H_Xma I _STR(F)	CCCGGGATGTCCATCTTCCTCATCGCA ACC
PM_F3’5’H_Xma I _STO(R)	CCCGGGTCAAACACCCCCATACGCCG
PM_F3’H_Xho I _STR(F)	CTCGAGATGATCGTCGTCAACTCCGCC
PM_F3’H_Xho I _STO(R)	CTCGAGTCACAAATAAGCCTCCGGTTC AAG
Delp_F3’5’H_Xma I _STR(F)	CCCGGGATGTCTATAAGCCTGTTCCTTG

	CTGG
Delp_F3’5’H_Xma I _STO(R)	CCCGGGTCAGACTACATATGCAGAGGG TGGC
5’ and 3’ RACE	
F3’5’H_RACE_3RA1	AGCCCTGCGAGGTAGAAGGTTACCAC
F3’5’H_RACE_3RA2	AAGACCTGGCTTTTGGTCAACATATGG GC
F3’5’H_RACE_5RA1	GTCACCAACAGCGGCACCG
F3’5’H_RACE_5RA2	TCCATGTCCAGCTCCACCACCCC
